# Nuclear localization and intensity of staining of nm23 protein is useful marker for breast cancer progression

**DOI:** 10.1186/1475-2867-8-6

**Published:** 2008-05-05

**Authors:** Nawfal I Ismail, Gurjeet Kaur, Hasnah Hashim, Mohammed S Hassan

**Affiliations:** 1 Advanced Medical and Dental Institute (AMDI), Universiti Sains Malaysia (USM), Penang, Malaysia

## Abstract

**Background:**

Breast cancer is the most common cause of cancer death in the western world. The expression differences of many proteins are associated with breast cancer progression or suppression. The purpose of the study was to determine the expression of nm23 protein in the invasion status and metastatic potential of breast cancer by using tissue microarray and to determine its role in breast cancer based on the expression of nm23 gene product.

**Method:**

nm23 protein expression was examined by immunohistochemistry (IHC) using commercially available tissue microarray containing malignant and normal breast tissues from 216 patients.

**Results:**

a similar percentage of cases showed positive cytoplasmic/nuclear staining for nm23 in normal breast tissue (85.7%), primary breast carcinoma node negative (97.5%) and carcinoma with lymph node metastasis (92.1%). Nuclear localization of staining for nm23 protein was higher in infiltrating ductal carcinoma (IDC) node positive (24.3%) and in matched lymph mode metastasis (18.9%) compared to IDC node negative (4.9%). Strong intensity of cytoplasmic/nucleus staining was observed in IDC node negative (42.6%), in IDC node positive (57.1%), and Infiltrating lobular carcinoma (ILC) node negative (44%) compared to normal breast tissue (16.7%).

**Conclusion:**

nm23 protein expression appears widely expressed in normal breast, early and advanced breast cancer stages. Interestingly our study found that strong staining intensity and nuclear localization of nm23 protein may prove to be a useful marker of breast cancer progression.

## Introduction

Cancer is characterized by uncontrolled growth (proliferation) of cells [1-3] and their ability to invade other organs or tissues, either by invasion or metastasis [2-4]. Although there are many types of cancer, all begin with uncontrolled growth of abnormal single cells in the body [3]. Breast cancer is the most common malignant neoplasm in women. Breast cancer is the commonest cancer among women in United Kingdom [5]. Breast cancer comprised 30.4 % of all female cancers in Malaysia in 2002 [6]. Approximately 182,000 estimated new cases of women with breast cancer are seen in the United States each year [7,8]. The number of women who die of breast cancer is approximately 46,000 each year in USA [7]. One out of eight women in United States and out of ten women in Europe is at risk of developing breast cancer at some point during her lifetime [9]. Most deaths of women with breast cancer arise not as a result of primary tumor but from its metastatic spread to distant sites in the body [10,11]. Once spread and secondary masses are formed, breast cancers are usually incurable [12]. Approximately one-third of the node negative breast cancer patients develop metastatic disease, whereas the other two-thirds do not develop metastasis despite not receiving chemotherapy [13]. Breast cancer is considered to be a systemic disease as most breast carcinoma metastasizes before diagnosis of the primary lesion [14]. Therefore, early detection of metastasized lesion and identification of more effective therapeutic modalities for metastatic disease are necessary if the prognosis for patients with advanced breast cancer is to improve. nm23 gene located on chromosome 17 q21.3 (A subunit by nm23-H1 and B subunit by nm23-H2) encodes nucleoside diphosphate kinase. It has been reported that differential regulation of nm23 by p53 in different cell types is an important component in the molecular mechanisms of tumor metastasis [15,16]. Other study reported that high nm23 expression is associated with older age (> 35 years) and smaller tumor size [17]. Also high nm23 expression is associated with the absence of distant metastasis [18]. Low expression is predictive of distant metastasis and appears to be a risk factor that is independent of the presence or absence of positive axillary nodes at diagnosis [18]. In contrast, several studies of lung and pancreatic carcinoma reported that nm23 expression correlated with advanced tumours [19-22]. Thus, the putative usefulness of nm23 in prediction of the clinical course of breast cancer patients remains to be identified. The main purpose of the study was to find the expression pattern of nm23 proteins in different types of breast cancer with or without lymph node involvement and to determine its role in breast cancer using tissue microarray.

## Materials and methods

Formalin-fixed paraffin-embedded tissue microarrays from 188 lymph node negative breast cancer patients, 50 breast cancer patients with lymph node metastasis (50 malignant tissues and 50 matched lymph node tissue cores with metastasis) and 8 normal breast tissue cores were analyzed by immunohistochemistry for the expression nm23 protein. Included in this study were patients with infiltrating ductal carcinoma, infiltrating lobular carcinoma, normal breast tissue and lymph node metastasis. The final number was 122 tissue cores of node negative infiltrating ductal carcinoma, 41 node negative infiltrating lobular carcinoma, seven normal breast tissue, 40 node positive (38 infiltrating ductal carcinoma, 2 infiltrating lobular carcinoma) and 46 tissue cores of lymph node metastasis. We did not evaluate infiltrating lobular carcinoma node positive because of the low sample size (2 cases). Forty six 3 tissue cores were excluded from statistics because very little cancer cells or no breast cancer tissue were seen. The total number after exclusion was 254 tissue cores (37 matched tissues) of 216 patients.

### Immunohistochemistry (IHC)

In principle, rabbit Anti-Human polyclonal primary antibody against nm23 protein (Code No. A0096, from DakoCytomation, Denmark) was used on deparaffinized tissue microarray slides (Cat. No. BR 2001, BR 1001 from Biomax, USA). A secondary detection system (DAKO Envision) enhanced with conjugated polymer was used to bind with the primary antibody. DAB chromogen was used for permanent color development and detection under microscope.

The percentage of carcinoma cells with cytoplasmic/membranous/nuclear staining was recorded on each specimen at 200× magnification, using light microscope. The expression of nm23 was scored in all tumors as positive or negative. Positive: ≥ 75% stained cells. Negative: no cells were stained. Also the intensity of staining was categorized into three groups: weak, moderate and strong. This was ascertained by a single qualified pathologist.

**Figure 1:**
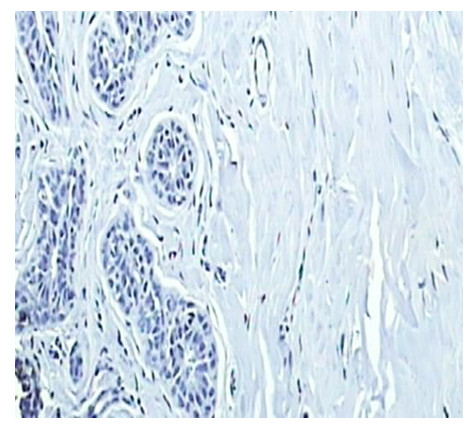
Negative control, nm23 protein immunohistochmical staining in normal breast tissue, showing absent staining (×200).

A tissue section of normal breast was used as positive control for nm23. Rabbit IgG isotype (Sigma- Aldrich, USA) was used instead of primary antibody in the immunohistochemical technique on a tissue section each of breast cancer and normal breast as negative control (Figure 1).

The tissue microarray slides were placed on hot plate at 60°C for 30 minutes. The slides were immersed in two changes of xylene (10 minutes for each) to remove paraffin. Slides were then immersed in 3 different concentrations of ethanol 100%, 95%, 70% ethanol for 5 minutes each. Slides were rinsed with distilled water to remove ethanol and left for 5 minutes. The slides were then placed in target retrieval solution EDTA buffer pH 9.0 (DAKO) and heated on microwave for 20 minutes at maximum setting. Slides were allowed to cool at room temperature and rinsed with Tris Buffered saline (TBS) mixed with tween 20 (2 changes). Slides were covered with peroxidase blocking solution (DAKO) and left for 15 minutes, followed by rinsing with TBS buffer mixed with tween 20 (2 changes). Then 200 μl of primary antibody (dilution 1:25) was added on the tissue microarray slides and left for 60 minutes, followed by rinsing with TBS mixed with tween 20 (three changes one minute each change). Two drops of DAKO Envision/HRP, Rabbit/Mouse (secondary antibody) were added on the slides and left for 30 minutes, followed by rinsing with TBS mixed with tween 20 (Three changes, one minute each change). After that DAB substrate (DAKO) was added on section slides and left for 10 minutes (40 μl DAB concentrate, 2 ml substrate buffer), followed by rinsing, immersion into hematoxylin and 4 different concentrations of ethanol 70%, 80%, 95% and 100% for 2 minutes each. After that slides were immersed in two changes of xylene (2 minutes each) and cover slipped. All incubation steps after heat induced epitope retrieval were carried at room temperature.

## Results

A majority of positive cases (i.e., ≥ 75 % stained cells) showed cytoplasmic stained cells with nm23 protein. The immunohistochemical expression of nm23 protein was observed in 97.5% (119/122) of node negative infiltrating ductal carcinoma (IDC). Strong intensity of staining was observed in 42.6% (52/122) and moderate intensity in 35.2% (43/122).

**Figure 2:**
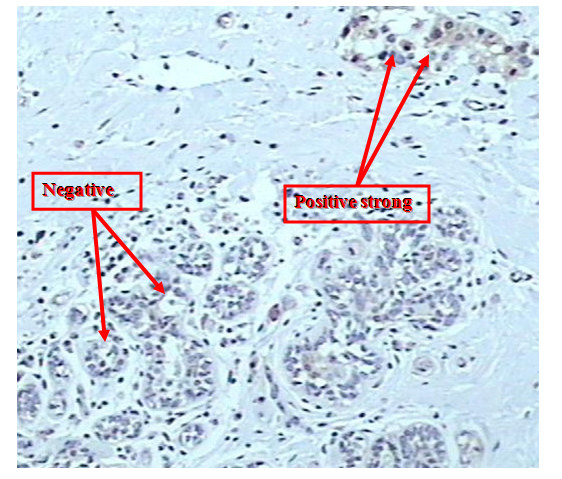
Strong staining intensity of nm23 protein within infiltrating lobular carcinoma while absent within adjacent normal breast tissue (×200).

**Figure 3:**
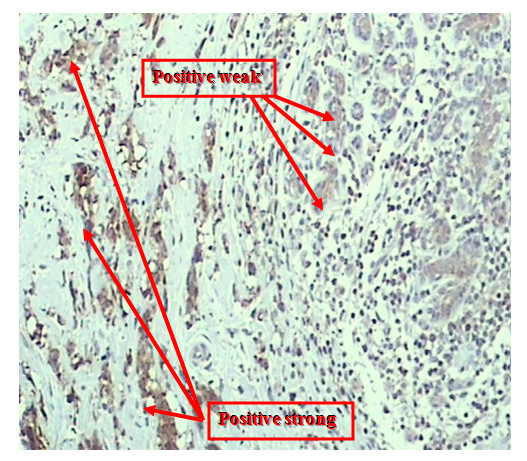
Strong staining intensity of nm23 protein within infiltrating lobular carcinoma while weak staining intensity within adjacent normal breast tissue (×200).

Positive expression of nm23 was observed in 61% (25/41) of node negative infiltrating lobular carcinoma (ILC). The strong staining intensity were observed in 44% (11/25) while moderate staining in 16% (4/25). The expression of nm23 protein in normal breast tissues was seen in 85.7% (6/7) cases. Weak staining intensity was observed in 66.7% (4/6) and strong staining in 16.7% (1/6). Different staining intensity had been found in (2/2) tissue cores containing infiltrating lobular carcinoma (strong staining intensity) and normal adjacent breast tissue (weak staining intensity) (figure 2, figure 3).

The staining of nm23 in node positive infiltrating ductal carcinoma (IDC) was observed in 92.1 % (35/38) with strong staining intensity noted in 57.1% (20/35) cases. The expression of nm23 protein was observed in 89.2% (41/46) of metastatic infiltrating ductal carcinoma (IDC with lymph node metastasis) with strong staining intensity noted in 31.7 % (13/41) cases.

It was observed that 89.2% (33/37) of the cases showed positive expression of nm23 protein in both infiltrating ductal carcinoma of breast and its matched lymph node metastasis. Two of 37 cases (5.4%) showed negative expression of nm23 in both sites of infiltrating ductal carcinoma and its matched lymph node metastasis. One case showed positive expression of nm23 at the site of infiltrating ductal carcinoma and negative nm23 expression in its matched lymph node metastasis. Also one case showed negative expression of nm23 infiltrating ductal carcinoma with positive nm23 expression in its matched lymph node metastasis (Table 1).

**Table 1:** The staining of nm23 in IDC and its lymph node metastasis for 37 patients

Staining	Primary site IDC	Matched lymph node
Both Positive	33(89.2 %)	
Both negative	2 (5.4 %)	
positive IDC only	1 (2.7 %)	0
positive Lymph node only	0	1 (2.7 %)
Total	37 (100%)	

**Figure 4:**
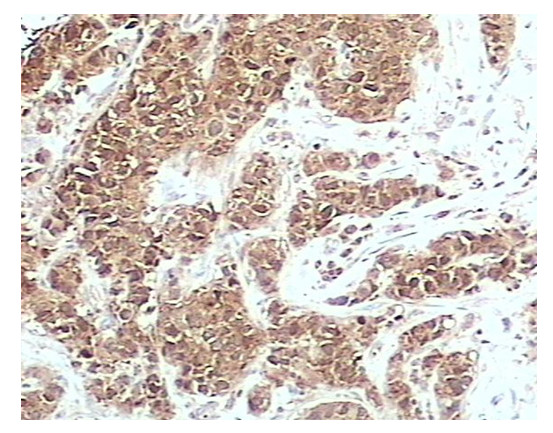
nm23 protein immunohistochmical staining in infiltrating ductal carcinoma, showing Strong nuclear and cytoplasmic staining (×200).

Both nuclear and cytoplasmic staining was seen in 6/122 (4.9%) of node negative IDC cases, 2/41 (4.8%) of node negative ILC cases, 9/37 (24.3%) of node positive IDC cases and 7/37 (18.9%) (Figure 4) in the matched lymph node metastasis cases (Table 2). Nuclear staining was not seen in normal breast

**Table 2:** Positive nuclear staining of nm23 in IDC, ILC node negative and positive

Site	Positive Nuclear staining	*n *(%)
IDC node negative	6	122 (4.9 %)
ILC node negative	2	41 (4.8 %)
IDC node positive	9	37 (24.3 %)
Matched Lymph node	7	37 (18.9 %)

## Discussion

nm23 protein was originally identified as a metastasis suppressor protein [23] and many previous studies reported reduced expression of nm23 protein correlated with metastasis or reduced survival for patients with breast cancer [24-29]. Recurrences of breast cancer were accompanied by nm23-loss [30]. In contrast, several studies of lung and pancreatic carcinoma reported that nm23 expression correlated with an advanced tumor [19-22]. Also several breast carcinoma studies have shown that low nm23 expression correlates with poor prognosis [31-33] but the dynamics of its expression pattern and compartmentalization in breast cancer remains poorly understood.

nm23 was expressed in normal breast tissue as well as in node negative IDC breast cancer, node negative ILC breast cancer, node positive IDC breast cancer, and matched lymph node metastasis in 85.7%, 97.5%, 61%, 92.1%, and 89.2% cases respectively. Among 97.5% node negative IDC cases, 42.6% had strong staining intensity and only 19.7 % had a weak staining. nm23 was expressed in 61% of node negative ILC cases indicating that nm23 protein expression was associated more with infiltrating node negative ductal carcinoma than node negative infiltrating lobular carcinoma. As expected, ILC has been reported to be more aggressive in nature compared to IDC. Expression of nm23 protein was observed in normal breast tissues in 85.7% of cases with strong staining intensity observed in only 16.7% cases. In two tissue cores it was clear that normal breast had much weaker or negative staining compared to adjacent lobular cancer cells. These results suggest nm23 expression increases in intensity in breast cancer compared to normal breast. Usually normal breast tissue expresses nm23 at baseline level in contrast to the strong expression which may reflect abnormal accumulation of nm23 (may be the altered form which is functionally inactive but resistant to degradation) in the early phases of cancer genesis.

In this study we observed a similar percentage of cases with positive staining for nm23 in normal breast tissue, node negative IDC as well as with lymph node metastasis with notable exception ILC. This suggests a relationship between a down regulation of nm23 expression and ILC. It may be possible that, other pathways are involved in progression of metastatic IDC.

However, nuclear staining together with cytoplasmic staining was observed in cancer cells in 6/122 (4.9%) of IDC node negative cases, 2/41 (4.8%) of ILC node negative cases, 9/37 (24.3%) of IDC node positive cases and 7/37 (18.9%) in matched lymph node metastasis cases. These results indicate that nuclear localization of nm23 protein is seen more frequently in node positive metastatic cases compared with IDC or ILC node negative cases, suggesting that abnormal nuclear accumulation of nm23 may provide an indication of tumor progression. We cannot be sure whether this nuclear accumulation is due to altered (mutated or truncated form) nm23 with different half-life as up-regulation of nm23 expression is unusual in at least metastatic carcinoma considering its role as tumor suppressor. However, this requires to be proven.

## Conclusion

In conclusion nm23 protein expression appears expressed widely in normal breast, early and advanced stages of breast cancer, predominantly in ductal carcinoma. It suggests a less significance of nm23 induced pathway of tumor suppression. While, in addition, abnormal cytoplasmic and nuclear localization of this protein in node positive and metastatic cases suggest a possible role of altered nm23 which is functionally inactive with longer half-life. However, this study indicates a complex role of nm23 in breast cancer of different types and in metastatic stages and may not solely function as tumor suppressor as commonly perceive. A larger study with more ILC and metastatic cases may clarify the role and function of nm23 in breast cancer.

## Abbreviations list

IHC: Immunohistochemistry; IDC: Infiltrating ductal carcinoma; ILC: Infiltrating lobular carcinoma.

## Authors' contributions

NII carried out Immunohistochemical part of the study and lab work, participated in drafting the manuscript. GK carried out the pathological part of the study, participated in drafting the manuscript. HH performed the statistical analysis. MSH initiated the project, participated in drafting the manuscript. All authors read and approved this manuscript.
